# The Soil Microbiota Recovery in the Agroecosystem: Minimal Information and a New Framework for Sustainable Agriculture

**DOI:** 10.3390/ijerph19095423

**Published:** 2022-04-29

**Authors:** Alessandro Bergna, Stephen J. Maund, Claudio Screpanti

**Affiliations:** Syngenta Crop Protection AG Research Chemistry, Schaffhauserstrasse 101, 4332 Stein, Switzerland; steve.maund@syngenta.com (S.J.M.); claudio.screpanti@syngenta.com (C.S.)

**Keywords:** soil health, agrochemicals, agroecosystem management, soil microbiome, microbiology, microbiome recovery

## Abstract

The efficient management of soil represents a mission of vital importance for meeting the continuously increasing agricultural demand in a sustainable way. Decades of research identified in the biotechnological potential of soil microorganisms an always more practicable channel for achieving these goals. Due to the complexity of soil microbial communities and their tight connection to soil characteristics, it is still difficult to define universal strategies for an efficient and sustainable agroecosystem management. We here propose a new framework for the assessment of the impact of agricultural practices in the agroecosystem that revolves around the concept of microbial community recovery. This assessment is based on the selection of (i) a representative temporal interval, (ii) a representative agricultural system and (iii) monitoring tools able to assess the expression levels of microbial functionality in soil. This approach can be especially valuable for evaluating the effects of agrochemicals and other agronomical amendments (of different nature: biological, physical, chemical) on the soil microbiota. In the same way precision-medicine tries to tailor drugs on an always smaller subset of patients’ characteristics, a new generation of agrochemicals can be developed and tested considering soil characteristics in order to minimize their off-target effects. What remains central in this paradigm is the promotion of Soil Health maintenance practices. As for healthy humans, a healthy soil is more resilient and tolerates treatments and stresses better while recovering more quickly.

## 1. Introduction

### 1.1. The Role of Soil Microorganisms for Soil Health

‘Our’ soil is a fundamental resource for the sustainment of every human activity. Nonetheless the management of this finite resource has never been more important than in our times. A continuously growing population is demanding access to safe food while our ability to efficiently produce it in a sustainable way remains limited. In view of these necessities, we want to propose a reconsideration of the role of agrochemicals with specific focus on the opportunity to sustain crop productivity while maintaining or improving soil quality.

This central ecological role of soil is largely due to its microbiota of extraordinary taxonomical and functional diversities. This second element is the key factor that allows the soil microbiota to be the engine of our ecosystem. Key ecosystem processes ascribable to soil microbial activity account for the regulation of carbon dynamics, the regulation of greenhouse gases (as hydrogen, carbon dioxide, nitric oxide, nitrous oxide, methane), the mitigation of soil erosion, the degradation of pollutants, the regulation of soil acidity and the mediation of the cycling of nutrients [1,2]. In this last process, all the elements crucial for plant and animal life sustainment undergo complex transformation cycles that take place in the soil and are directly modulated by soil microorganisms. Of primary interest is how multiple bacterial, fungal or archaeal taxa are required for the execution of these cycles. From nitrogen to phosphorus and carbon, all these cycles require multiple reactions that transform non-bioavailable molecules to bioavailable forms assimilable by plants, animals and other microorganisms. Virtually no single microorganism possesses the genetic/enzymatic equipment to perform a complete cycle. This is the reason why the most important indicators of a healthy soil are often not single microbial taxa but rather the presence of a microbial network with complementary genetic/enzymatic tools. These bacterial, fungal, and archaeal species represent the microbial networks that allow soil health related functions to take place.

### 1.2. Perturbations in the Soil Microbiota

As proof that we may have domesticated our crops but not our ecosystem, a large part of anthropogenic activities connected with crop production mines the functional state of our soils [3] at both the macro and micro scale. Due to their functional specificity and their pivotal role in soil, microorganisms are becoming biosensors of central importance for soil health [4]. 

Soil microbial communities in the agroecosystem are normally structured around the interplay between several biotic and abiotic factors that are the primary determinant of soil microbiota composition and functioning. These key factors are soil pH, the quality and quantity of organic carbon, nitrogen availability, temperature and redox status [1,5,6,7,8,9]. 

In the context of a system already deeply modified by these factors, agricultural practices can add a second level of modifications to the soil microbial assemblage by directly interacting with the microorganisms or by modifying the already mentioned soil properties. An example is the choice of the crop genotype and of crop rotation. Cultivar-specific factors like plant root morphology or the quantity and quality of root exudates can influence the recruitment of plant growth promoting rhizobacteria (PGPR) and arbuscular mycorrhizal fungi (AMF) [3,10,11,12,13,14,15]. Soil physical disturbances can also modify the soil microbiota. Tillage practices can affect both soil microbiota composition and functionality to different degrees depending on soil physiochemical properties [16] by changing the physical status of soil microhabitats and disrupting the fungal hyphal networks [16,17] and resulting in the reduction of the abundance of soil bacteria such as *Alphaproteobacteria* and *Actinobacteria* [18]. Similarly, the use of mineral fertilization and use of crop protection products can restructure the soil microbiota [19]. For instance, intensive N management strategies can modify the soil pH resulting in modifications in the dominant taxa of the soil microbiota and the richness of *Actinobacteria* [20]. Nonetheless, also this effect can vary depending on environmental and crop management related factors [3,21].

### 1.3. The Soil Microbiota and Chemical Disturbances in the Agronomical Scenario

The interaction between agrochemicals and the soil microbiota must represent a central theme for the new generation of disease control products. In addition, well-designed experiments focusing on long-term effects in the agricultural environment are needed [19]. In the study of disturbance ecology, ‘time’ and ‘scenario of investigation’ are key parameters needed to understand the ecological consequences of the disturbance [22]. The study of agrochemicals effects is not exempt [23].

Timescale of investigation. As defined by Graham and colleagues, “a single type of event may constitute a disturbance at one timescale, but not at another” [22]. In other words, if a treatment would affect microbially mediated soil functions, a short-term study would observe a strong community disturbance, while a long-term study may also observe the recovery of the temporarily loss function. In the same way, a short-term study based on a single agrochemical application may overlook potential press disturbances connected with its accumulation in soil. Long-term studies would therefore allow us to understand key ecological parameters of community dynamics as distinguishing between pulse-disturbances and press-disturbances while evaluating community stability, resistance/sensibility, and resilience.

The agroecosystem. In presence of a complex system in which biotic and abiotic factors are the main drivers of soil microbial community structure, also the activity and degradation of agrochemicals depend on factors and processes connected with soil characteristics [19]. The soil physical and chemical state, the processes of adsorption and desorption, the interaction with the plant (plant uptake), processes of volatilization, photolysis and chemical conversion [19,24] may all modify the effect of agrochemicals on microbial communities and its stability in soil [25]. Depriving the studies of their agronomical scenario and bounding them to mesocosm studies [26] may have the effect of focusing on direct-toxicity effects that are not indicative, while potentially hiding off-target effects that require a more complex system for their study.

### 1.4. Soil Functional Profile and Functional Diversity as Key Determinant of Soil Microbial Community Disturbance

Virtually every agricultural practice can have an impact on soil microorganisms. In order to fully explore the biotechnological potential of the soil microbiota as a tool for sustainable and regenerative agriculture, we need to quantitatively distinguish and compare between positive and negative effects. 

A parameter commonly used to evaluate the impact of disturbances on microbial communities is microbial diversity. This is commonly due to the assumption ‘more taxa equal more functions’ that doesn’t always prove to be true. Indeed, taxonomical diversity can represent a bad predictor of functional diversity. This is mainly due to our only partial ability to correctly predict functions from genetic content [27,28], to understand the nature of inter-microbial interactions [29], and to the extraordinary degree of functional redundancy present in soil. While functional redundancy could act as an insurance for the community in presence of prolonged stresses [30], it can also represent an obstacle when interpreting the ecological impact of eliminated taxa (since other microorganisms could take over the otherwise loss function).

In addition to these more methodological aspects, the relevance of taxonomical diversity for the prediction of soil ecosystem functioning is debatable also from the ecological standpoint. A clear example is the application of plant-growth-promoting-microorganisms as agronomical amendment. The inoculation of these beneficial microorganisms can successfully promote plant health and nutrient geochemical cycles in soil [31]. On the other hand, these new taxa can dominate the newly established microbial community, resulting in a relative abundance reduction of keystone microorganisms and of overall taxonomical diversity [32]. 

For all these reasons, basing our observation of microbial community shifts on taxonomy-based indexes might not return a clear picture of the system functioning state and of ecosystem functioning.

**Figure 1:**
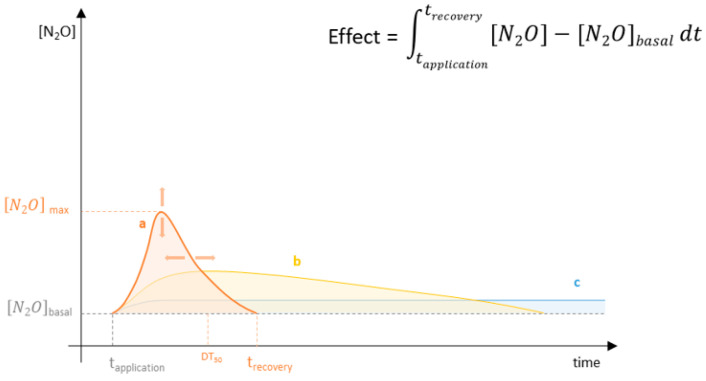
Representation of the monitoring over a long-time period of three hypothetical greenhouse gas emission profiles associated to different stress events (a, b and c). As represented with the arrows on the event ‘a’, environmental conditions and soil properties can modify the effect of agrochemicals on microbial communities’ functions and their recovery time. The short-term assessment of each of these events may detect the acute effect on the emission of greenhouse gasses ([N_2_O]_max_) but would not be able to predict the environmental impact of the events. The event ‘a’ and ‘b’ can have a similar environmental impact in spite of ‘a’ having a considerably higher short time impact but a faster recovery to basal emission levels. Conversely, the event ‘c’ is characterised by a lower environmental impact in the short but also long timeframe. This event would likely require an anthropogenic intervention to restore the basal level of greenhouse gas emission.

**Figure 2:**
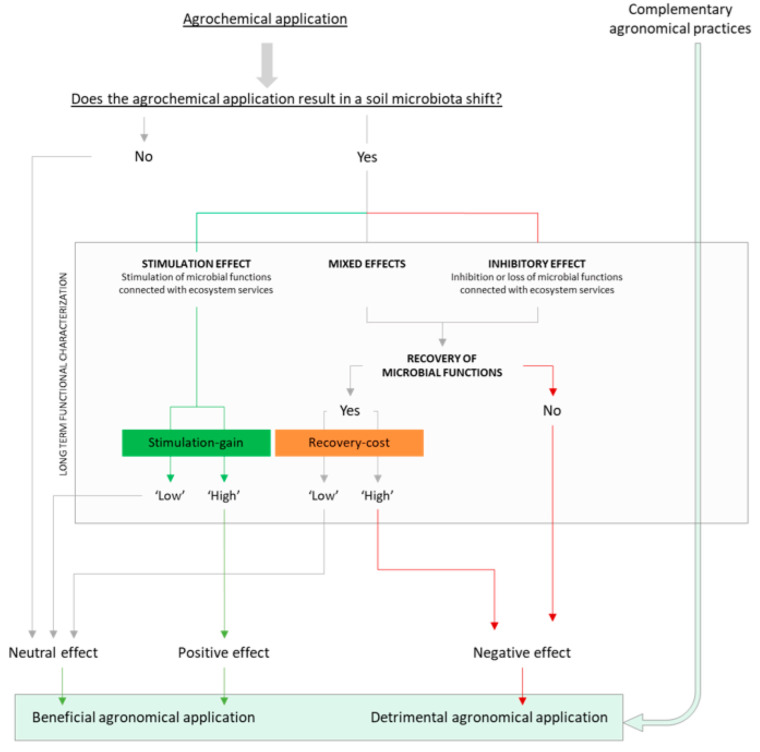
Model for the assessment of soil microbiota disturbances and in the agroecosystem.

Even if taxonomical diversity must be still regarded as an important ecosystem parameter, the prolonged assessment of functional diversity would allow to highlight the microbial networks that are in a pristine and functioning state without relying on predictive tools. In addition, an extended set of time-points would allow the quantification of functionality inhibition and of phenomena of paramount importance like functional recovery and functionality loss (otherwise difficultly predicted with genomic tools). These phenomena become of central importance in complex systems as soils where physical-chemical characteristics have a great impact on both the structure of the microbiota and the behaviour of the agrochemical in soil (Figure 1). For this reason, in addition to the necessity to test the effect of agrochemicals over time, it would be advisable to test with a set of representative soils encompassing the main microbiota-modulating conditions. To evaluate and compare the effect of agrochemicals on microbially mediated functions, we here propose a roadmap that follows an empirical line of reasoning (schematised in Figure 2). 

Following an agrochemical application, a shift in the microbial community could occur. In presence of no substantial microbial community shifts (neutral effect), the application can be considered to have an overall positive effect on the agroecosystem. In fact, a non-altering agrochemical application would have productivity-enhancing effects on the target crop without causing off-target effects. 

In presence of a shift in the soil microbial community, a characterisation of soil functional state and of key soil health indicators can be used to assess whether microbially mediated soil ecosystem functions have been stimulated (positive effect), inhibited (negative effect) or both (mixed effect). While in the presence of stimulating effects the benefit is self-evident, in presence of negative and mixed effects our line of reasoning diverges from most studies. 

As previously stated, in absence of a timescale the inhibition of microbially mediated functions is not sufficient to define the event as a disturbance [22]. In fact, only an assessment able to monitor and record the evolution of these functions over time could observe whether the microbiota would spontaneously recover the inhibited functions (as in the curves ‘a’ and ‘b’—Figure 1), and how long it would take, or if they would permanently be lost (as in the curve ‘c’). With this proposed approach it is possible to evaluate the trade-off of the application by comparing the ‘recovery-debt’ associated with inhibiting events [33] and the ‘beneficial-gain’ associated with stimulating events. Once again, with the example provided in Figure 1 we can observe how, even if the negative consequences associated with the event ‘c’ would appear minimal in the short-term scenario, the functionality loss would persist over time and would dramatically increase the damage.

The microbiota shift can therefore be expressed as a ‘cost’ for the agroecosystem and the effectiveness of the agronomical amendment can be compared with the lawmaker recommendations and with alternative agronomical practices. For example, agronomical strategies based on the application of herbicides and no-till could be compared with tillage-based strategies. 

## 2. A Minimal Information Model for Soil Microbiota Disturbance Assessment

Due to the complexity of soil-plant-microbe interactions we acknowledge the difficult task of the lawmaker for developing universal guidance. At the same time, the scientific community should be aligned and focus on clarifying the system without simplifying it. In order to assess the impact of agronomical practices on microbially mediated biogeochemical cycles and services, we here include a list of recommendation that would allow the establishment of a comprehensive study.

### 2.1. Methodology

As we have described, physical and chemical characteristics of soils are the main determinants of the soil microbial assembly and of its resilience. They often represent a driving force of higher hierarchy compared to biological factors. In addition, our understanding of soil microbial communities and of microbiota dynamics is still partial. For these reasons, we suggest moving beyond the description of community composition in favour of the microbial functional diversity by assessing community functional outputs with classic techniques. Only in this way it becomes possible to study the active microbial networks.

Omics techniques are methodologies of exquisite power for studying microbial communities and predicting their functional potential [34]. Due to the predictive nature of these technologies, -omics techniques can be considered critical for system modelling and predictive studies. Among these, amplicon sequencing (also known as “metabarcoding”) [35] and shotgun metagenomics are -omics techniques being used for surveys of soil microbial communities [36,37]. While the information regarding the presence/absence of microbial taxa or genes gives the possibility to monitor microbial shifts in soil, in order to link these observations to microbially mediated processes in the soil, a monitoring of soil microbial activity is still required [23,38].

### 2.2. Soils of Selection and Reference Ecosystem

Given the importance of soil characteristics for soil microbiota setup, the soils selected for the study should be representative of the physical and chemical characteristics of the agroecosystem in which the application is foreseen. These characteristics are pH, organic carbon quality and quantity, bioavailability of nitrogen and phosphorous, moisture level, structure and temperature. 

Another factor to be considered is that agrochemicals are meant to be applied on the agroecosystem with the clear aim to sustainably protect crops. While studying soils in an undisturbed state would allow assessing the anthropogenic impact of agriculture on the environment, the influence of agrochemicals on soil microbial communities needs to be studied within the agroecosystem. In the current geopolitical state food production represents a key priority. It is therefore difficult to foresee that cultivated land would be reconverted in pristine natural ecosystem. For this reason, a comparison between the microbiota functioning state in pristine soils and agricultural soils is incorrect.

### 2.3. Time

Time is the key parameter that allows to define the microbiota shift as a disturbance. For this reason, it is advisable to adopt a monitoring-time that would encompass the time needed for the degradation of the agrochemical and the observation of an eventual microbiota recovery. An indicator of chemicals stability in each different soil is the DT_50_. The selection of the assessment time should therefore take in consideration this value. In addition, for a more comprehensive and univocal generation of data, the assessment time could also be expressed in function of this value. For example, for an agrochemical/soil combination in which the DT_50_ is 30 days, an assessment 60 days after application would be indicated as ‘2 ∙ DT_50_’ while an assessment 50 days after application can be indicated as ‘1.67 ∙ DT_50_’. With such parameter (instead of “assessment n days after application”) it would be possible to universally recognise whether the study considers a short- medium- or long-term observation.

## 3. Not Much Different from a Visit at Your GP

While this line of thinking might appear one sided, a critical analysis would understand that a pillar of our society like medicine is established on the same trade-off principle. 

The side effects of pharmaceutical drugs in humans and animals are well known by scientists, medical practitioners and, presumably, by the entirety of the consumers. Following the roadmap presented, the effects of medication drugs on the organism and on the human microbiota would often fall in the mixed effects category. Similarly to the variable effect of agrochemicals within the agroecological scenario, the extent of harm of xenobiotics on the human microbiome can vary among sex, existing medical conditions and inter- and intra-individual genetic variations [39,40]. What is more, a 2018 screening of commercial drugs highlighted how a consistent proportion of non-antibiotics drugs had inhibition effects on representative gut bacteria [41]. 

Yet, the use of pharmaceutical drugs is rooted in our society due to the evident effect that these medications have on human health. In other words, a cost-benefit analysis is convention and the recovery becomes a key parameter while side effects are factors to be studied and limited. Conversely, a large part of the world population takes food security for granted and neglects the impact that agricultural loss to pests (annual agricultural loss to pest estimated at 40% by FAO in 2017.) has on today’s growing population. These are likely the reasons why several bodies propose to ban agrochemicals even in absence of alternative management practices and even if modern agrochemicals have structures, physical properties and a design similar to pharmaceutical drugs [42]. While we acknowledge that the uncontrolled use of agrochemicals can affect soil functioning, neglecting the importance of soil microbiota recovery in the agroecosystem represents an evaluation mistake and a loss of opportunity.

## 4. Conclusions

Releasing the biotechnological potential of the soil microbiota represents one of the practicable solutions to produce food more efficiently and sustainably. We here proposed a new framework for the assessment and the monitoring of the interactions between agrochemicals and the soil microbiota within the agroecosystem. Soil microbial functioning state represents the key parameter in this assessment and allows to understand whether an agricultural amendment (chemical, physical or biological) may represent an opportunity or a threat. Moreover, in order to comprehensively assess potential disturbances, it is of upmost importance to place each assessment in the right time scale.

What remains central in this paradigm is the promotion of Soil Health maintenance practices. As for healthy humans, a healthy soil is more resilient and tolerates treatments and stresses better while recovering more quickly. ‘Precision medicine’ indicates all the efforts to better define diseases so to develop therapies targeting an always smaller subset of patients [43]. This would allow the development of therapies with enhanced effectiveness and lowered side effects. Similarly, a new generation of agrochemicals can be developed and tested accounting the variability of their effects on different soils while minimizing their off-target effects.
